# Supplementary material for: Transcriptional, epigenetic and metabolic signatures in cardiometabolic syndrome defined by extreme phenotypes
Source: Clin Epigenetics. 2022 Mar 12;14:39. doi: 10.1186/s13148-022-01257-z (PMC8917653; doi:10.1186/s13148-022-01257-z)
Supplement: Supplementary file 1 — Additional file 1: Fig. S1. Overview of experimental design. A. Parameters used to define lean individuals amongst blood donors and controls. B. Overview of the analysis set-up to determine the effects of bariatric surgery. To minimise batch effects, 20 additional blood donors ("Controls") were recruited and were not previously present in the original BluePrint cohort. We applied a set of filters (METHODS) to identify low risk individuals within the 20 Controls, designed hereafter as "Lean-Control". C. Overview of multi-omics integration. In brief, data from 202 individuals identified as blood donors ("BD") in the present study, were collected from BluePrint consortium. It included, for monocytes and neutrophils, H3K27ac ChIP-seq, RNA-seq and 450K methylation arrays. Additionally, we obtained anthropometric measurements (BW, BMI) and generated plasma biochemistry assays, plasma metabolomics, plasma lipidomics on samples collected from the same individuals at the same time. Case groups were composed of obese individuals referred for bariatric surgery and lipodystrophy patients. Data for these groups were generated in this study. We applied a set of filters (METHODS) to identify low risk individuals within the 202 blood donors, designed hereafter as "Lean-BD". [file 13148_2022_1257_MOESM1_ESM.pdf]

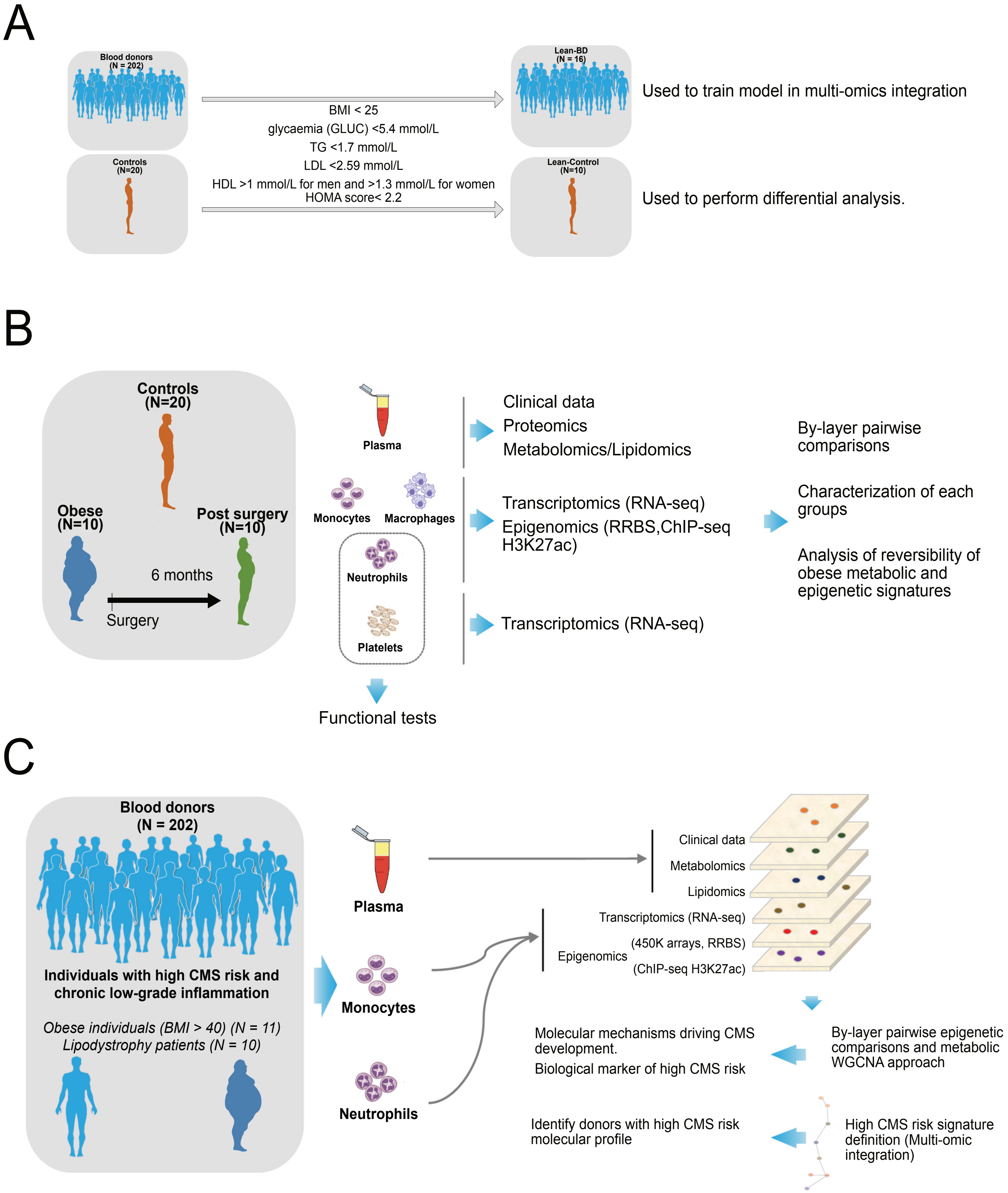

Supplementary figure 1. Overview of experimental design.

A. Parameters used to define lean individuals among blood donors and controls.

B. Overview of the analysis set-up to determine the effects of bariatric surgery. To minimize batch effects, 20 additional blood donors ("Controls") were recruited and were not previously present in the original BluePrint cohort. We applied a set of filters (METHODS) to identify low risk individuals within the 20 Controls, designed hereafter as "Lean-Control".

C. Overview of multi-omics integration. In brief, data from 202 individuals identified as blood donors ("BD") in the present study, were collected from BluePrint consortium. It included, for monocytes and neutrophils, H3K27ac ChIP-seq, RNA-seq and 450K methylation arrays. Additionally, we obtained anthropometric measurements (BW, BMI) and generated plasma biochemistry assays, plasma metabolomics, plasma lipidomics on samples collected from the same individuals at the same time. Case groups were composed of obese individuals referred for bariatric surgery and lipodystrophy patients. Data for these groups were generated in this study. We applied a set of filters (METHODS) to identify low risk individuals within the 202 blood donors, designed hereafter as "Lean-BD".
