# Supplementary material for: Transcriptional, epigenetic and metabolic signatures in cardiometabolic syndrome defined by extreme phenotypes
Source: Clin Epigenetics. 2022 Mar 12;14:39. doi: 10.1186/s13148-022-01257-z (PMC8917653; doi:10.1186/s13148-022-01257-z)
Supplement: Supplementary file 2 — Additional file 2: Fig. S2. Related to Figure 1—WGCNA analysis with BD individuals metabolite values and cluster functional annotation. A. Heatmap of BD individuals eigen-metabolites adjacencies in the consensus eigen-metabolites network. Each row and column correspond to one eigen metabolite (labelled by consensus module colour). The heatmap is colour-coded by adjacency, yellow indicating high adjacency (positive correlation) and blue low adjacency (negative correlation) as shown by the colour legend. B. Beeswarm plot using average eigen metabolites per cluster. Colours indicate cohorts. [file 13148_2022_1257_MOESM2_ESM.pdf]

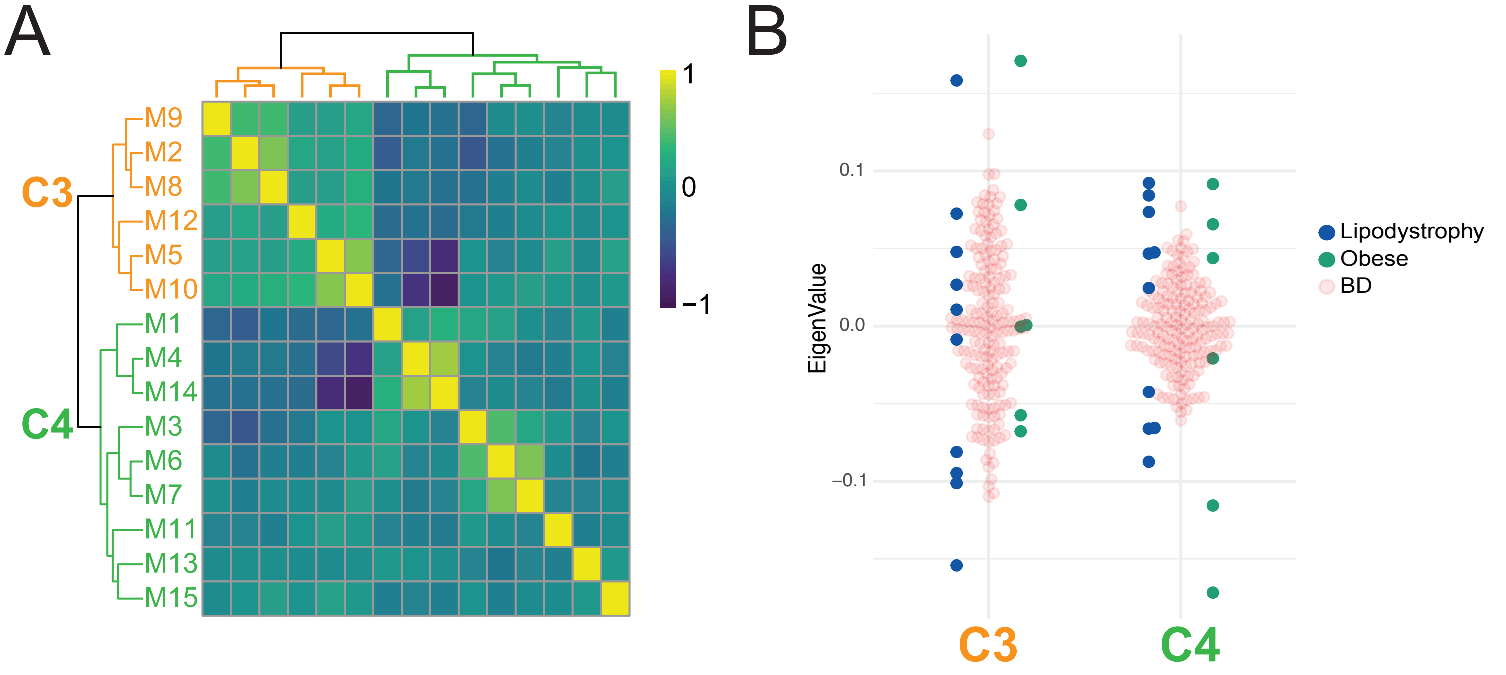

Supplementary figure 2 - Related to Figure 1 - WGCNA analysis with BD individuals metabolite values and cluster functional annotation.

A. Heatmap of BD individuals eigen-metabolites adjacencies in the consensus eigen-metabolites network. Each row and column correspond to one eigen-metabolite (labeled by consensus module color). The heat-map is color-coded by adjacency, yellow indicating high adjacency (positive correlation) and blue low adja-cency (negative correlation) as shown by the color legend. B. Beeswarm plot using average eigen-metabo-lites per cluster. Colors indicate cohorts.
