# Supplementary material for: Transcriptional, epigenetic and metabolic signatures in cardiometabolic syndrome defined by extreme phenotypes
Source: Clin Epigenetics. 2022 Mar 12;14:39. doi: 10.1186/s13148-022-01257-z (PMC8917653; doi:10.1186/s13148-022-01257-z)
Supplement: Supplementary file 3 — Additional file 3: Fig. S3. Related to Figure 2—Summary plots of different feature numbers in all comparisons. Barplots showing the number of features significantly different for each comparison in H3K27ac distribution (ChIP-Seq), gene expression (RNA-Seq) and DNA methylation (RRBS). Each bar is colour coded to represent the different cell types. [file 13148_2022_1257_MOESM3_ESM.pdf]

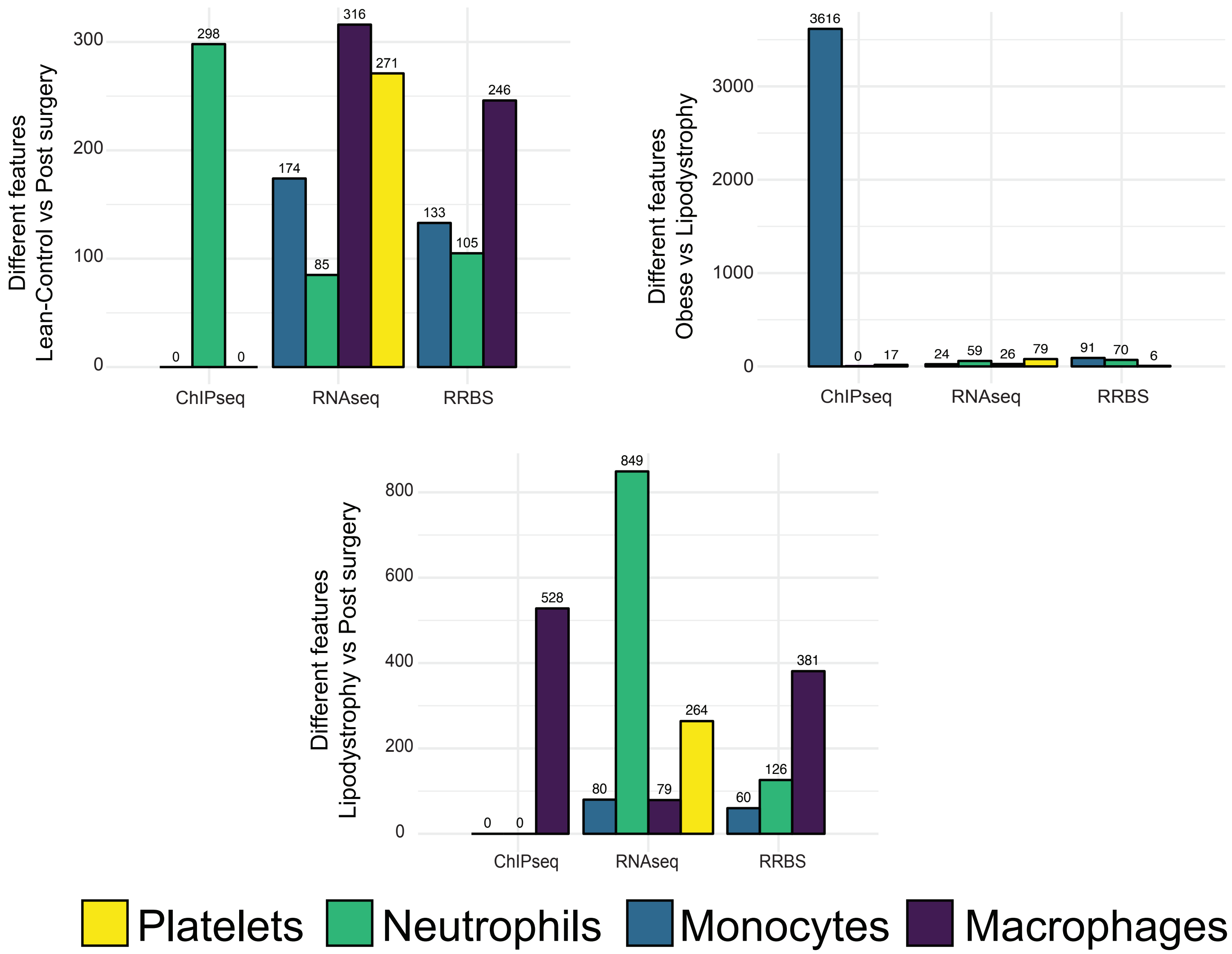


**Supplementary figure 3 - Related to Figure 2 - Summary plots of different feature numbers in all comparisons.**

Barplots showing the number of features significantly different for each comparison in H3K27ac distribution (ChIP-Seq), gene expression (RNA-Seq) and DNA methylation (RRBS). Each bar is color coded to represent the different cell types.
