# Supplementary material for: Transcriptional, epigenetic and metabolic signatures in cardiometabolic syndrome defined by extreme phenotypes
Source: Clin Epigenetics. 2022 Mar 12;14:39. doi: 10.1186/s13148-022-01257-z (PMC8917653; doi:10.1186/s13148-022-01257-z)
Supplement: Supplementary file 4 — Additional file 4: Fig. S4. Related to Figure 3—Multi-omic signatures of extreme phenotype groups and their use in prediction. A. Plots showing individuals ranked by their predicted probability of belonging to the obese group. As in Figure 3C, but for the Methylation (monocytes), RNA-Seq (monocytes), Metabolites, and ChIP-Seq (monocytes) data layers. B. Multi-omic model trained using lipodystrophy patients often predicts obese individuals to belong to the lipodystrophy group. As in Figure 3C (final plot), but training the multi-layer model using the Lipodystrophy and Lean-BD groups (rather than the Obese and Lean-BD groups). Using this model, Obese individuals were often predicted as belonging to the Lipodystrophy group. [file 13148_2022_1257_MOESM4_ESM.pdf]

A

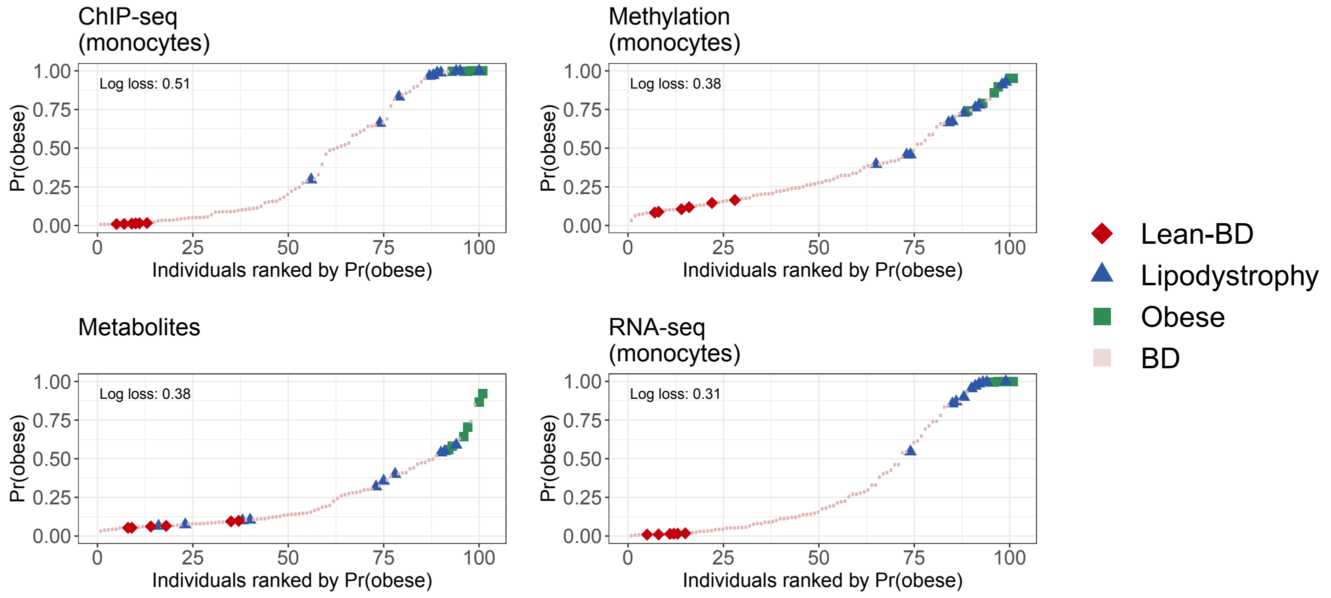

B

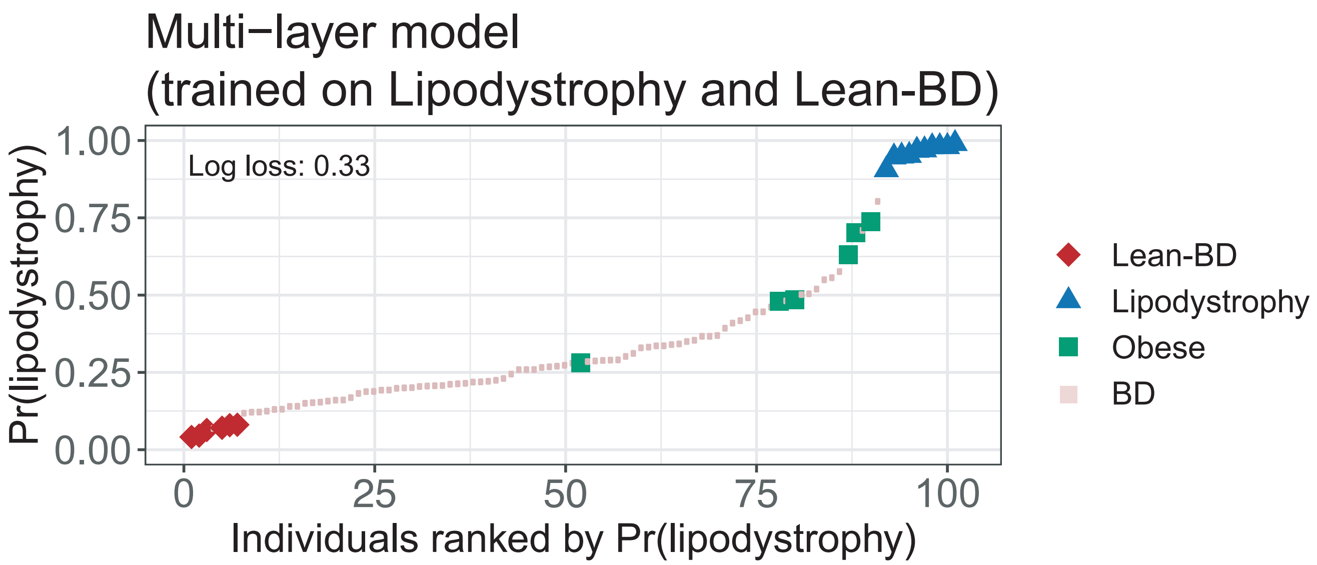

Supplementary figure 4 - Related to Figure 3 - Multi-omic signatures of extreme phenotype groups and their use in prediction.
A. Plots showing individuals ranked by their predicted probability of belonging to the obese group. As in Figure 3C, but for the Methylation (monocytes), RNA-Seq (monocytes), Metabolites, and ChIP-Seq (monocytes) data layers. B. Multi-omic model trained using lipodystrophy patients often predicts obese individuals to belong to the lipodystrophy group. As in Figure 3C (final plot), but training the multi-layer model using the Lipodystrophy and Lean-BD groups (rather than the Obese and Lean-BD groups). Using this model, Obese individuals were often predicted as belonging to the Lipodystrophy group.
