# Supplementary material for: Transcriptional, epigenetic and metabolic signatures in cardiometabolic syndrome defined by extreme phenotypes
Source: Clin Epigenetics. 2022 Mar 12;14:39. doi: 10.1186/s13148-022-01257-z (PMC8917653; doi:10.1186/s13148-022-01257-z)
Supplement: Supplementary file 5 — Additional file 5: Fig. S5. Related to Figure 3—A common pattern of associations between the prioritised lipid species and known CMS risk factors. The pattern of association between the prioritised lipids and known CMS risk factors in the NASH cohort (NASH cohort; left) agrees with the results from the present study (BD cohort; right). [file 13148_2022_1257_MOESM5_ESM.pdf]

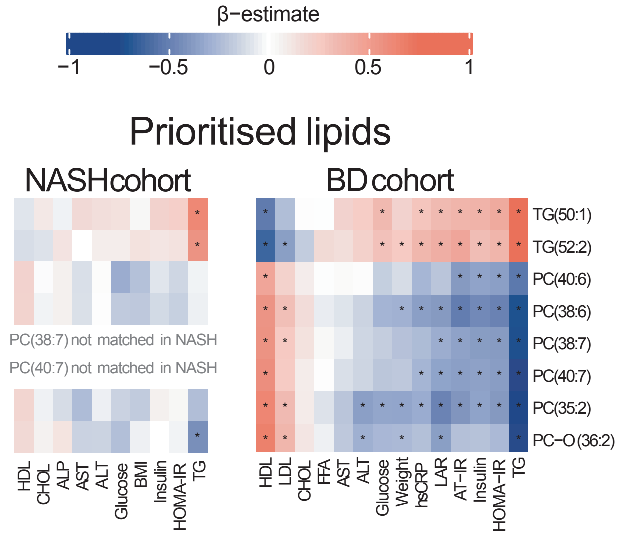

Supplementary figure 5 - Related to Figure 3 - A common pattern of associations between the prioritised lipid species and known CMS risk factors.

The pattern of association between the prioritised lipids and known CMS risk factors in the NASH cohort (NASH cohort; left) agrees with the results from the present study (BD cohort; right).
